# *“Getting the water-carrier to light the lamps”*: Discrepant role perceptions of traditional, complementary, and alternative medical practitioners in government health facilities in India

**DOI:** 10.1016/j.socscimed.2016.08.038

**Published:** 2016-10

**Authors:** K. Lakshmi Josyula, Kabir Sheikh, Devaki Nambiar, Venkatesh V. Narayan, T.N. Sathyanarayana, John D.H. Porter

**Affiliations:** a Indian Institute of Public Health, Hyderabad, Public Health Foundation of India, Plot # 1, A N V Arcade, Amar Co-operative Society, Kavuri Hills, Madhapur, Hyderabad, 500033, India; b Public Health Foundation of India, Plot No. 47, Sector 44, Institutional Area, Gurgaon, 122002, India; c London School of Hygiene and Tropical Medicine, Keppel St, London, WC1E 7HT, United Kingdom

**Keywords:** India, Traditional, Complementary, and Alternative Medicine, AYUSH, Mainstreaming, Pluralistic health system, Role perceptions, Role ambivalence, Integration

## Abstract

The government of India has, over the past decade, implemented the “integration” of traditional, complementary and alternative medical (TCAM) practitioners, specifically practitioners of Ayurveda, Yoga and Naturopathy, Unani, Siddha, Sowa-rigpa, and Homoeopathy (collectively known by the acronym AYUSH), in government health services. A range of operational and ethical challenges has manifested during this process of large health system reform. We explored the practices and perceptions of health system actors, in relation to AYUSH providers' roles in government health services in three Indian states – Kerala, Meghalaya, and Delhi. Research methods included 196 in-depth interviews with a range of health policy and system actors and beneficiaries, between February and October 2012, and review of national, state, and district-level policy documents relating to AYUSH integration. The thematic ‘framework’ approach was applied to analyze data from the interviews, and systematic content analysis performed on policy documents.

We found that the roles of AYUSH providers are frequently ambiguously stated and variably interpreted, in relation to various aspects of their practice, such as outpatient care, prescribing rights, emergency duties, obstetric services, night duties, and referrals across systems of medicine. Work sharing is variously interpreted by different health system actors as complementing allopathic practice with AYUSH practice, or allopathic practice, by AYUSH providers to supplement the work of allopathic practitioners. Interactions among AYUSH practitioners and their health system colleagues frequently take place in a context of partial information, preconceived notions, power imbalances, and mistrust. In some notable instances, collegial relationships and apt divisions of responsibilities are observed. Widespread normative ambivalence around the roles of AYUSH providers, complicated by the logistical constraints prevalent in poorly resourced systems, has the potential to undermine the therapeutic practices and motivation of AYUSH providers, as well as the overall efficiency and performance of integrated health services.

## Highlights

• The roles of AYUSH (TCAM) providers in public facilities in India are perceived discrepantly.
• Work sharing is dissimilarly interpreted by different health system actors.
• Interactions among practitioners of different systems of medicine are fraught.
• Logistical, infrastructural, technical, and administrative provision for TCAM is low.
• Policy and budgetary support for TCAM are inadequate for field development.

## 1 Background

Efforts to include traditional, complementary and alternative medical (TCAM) systems in the public health mainstream have been gaining momentum across the world (Lakshmi et al., 2015), particularly in developing countries, with the goals of enhancing populations' access to healthcare, optimizing the roles of healthcare providers, and promoting the different systems of medicine. The World Health Organization's traditional medicine strategy acknowledges the widespread use, accessibility, and cultural relevance of TCAM, advocates the inclusion of TCAM in public health systems for disease control and health promotion (WHO, 2002), and promotes the integration of TCAM in national healthcare systems (WHO, 2013). Many countries, such as China (Jingfeng, 1988), South Korea (Son, 1999), and Cuba (Appelbaum et al., 2006) have articulated national and sub-national policies for the integration of certain systems of TCAM into health service delivery, and for the provision and regulation of medical education, accreditation, licensing, and drug-regulation. A WHO global survey revealed that 32 percent of respondent countries had issued national policies on TCAM, and that 56 percent of the rest were in the process of developing such policies (WHO, 2005).

The Ministry of Health and Family Welfare of the Government of India comprised an autonomous unit tasked with regulation, education, accreditation, and provision for government-endorsed TCAM systems. This unit, originally established as the Department of Indian Systems of Medicine and Homoeopathy in 1995, was renamed the Department of AYUSH in 2003, and governed the provision and practice of Ayurveda, Yoga and Naturopathy, Unani, Siddha and Sowa-Rigpa, and Homoeopathy (AYUSH) in India. It was elevated to a Ministry in November 2014. A Draft National Policy on AYUSH is in development in 2016 (Ministry of AYUSH, 2015).

The National Rural Health Mission (NRHM), 2015, launched by the government of India in 2005, emphasized the “mainstreaming of AYUSH” as a strategy to increase healthcare access for the population, and to provide AYUSH providers with a platform to practise their systems of medicine in India (Department of AYUSH, 2011). This initiative included the appointment of AYUSH providers in public health facilities, in some instances, to work alone, and in many cases, to work alongside allopathic practitioners (in an arrangement termed ‘co-location’), as well as the involvement of AYUSH providers in national health programmes, such as those for the prevention and control of polio, tuberculosis, and malaria. These policies at the national level were then interpreted and implemented by the states. The establishment of new AYUSH facilities at healthcare centres at district and sub-district levels, and the upgradation of AYUSH facilities in hospitals and dispensaries, have been accomplished under the NRHM, in addition to the contractual appointment of approximately 11478 medical practitioners and 4894 para-medical workers across the country (Press Information Bureau, 2013).

Over the years, the integration of AYUSH providers into the public health system of India has proceeded in different ways, and to varying extents in the different states of India, partly due to different state interpretations of the policies. Integration as policy and health systems reforms requires attention to health goals and stakeholder roles, multi-level reform, and a reorientation of systems values (Sheikh and Nambiar, 2011). Reports from various states reveal numerous challenges, including shortfalls in recruitment and deployment of personnel, delayed or inadequate drug supply, insufficient infrastructure and personnel support, and problematic administrative structures and interpersonal interactions, in the mainstreaming of AYUSH (Chandra, 2011, SEDEM, 2010, Priya and Shweta, 2010, Lakshmi, 2012, Gopichandran and Kumar, 2012).

We conducted a study in three states of India to examine the operational and ethical challenges of AYUSH mainstreaming. The integration of the different systems of medicine in the public health system has at its centre the practitioners of the different systems of medicine. This paper presents findings on health policy and system actors' practices and perceptions related to AYUSH providers’ roles in government health services.

## 2 Methods

### 2.1 Research design

The protocol for this study received ethics approval from the Institutional Ethics Committee of the Public Health Foundation of India. The study was conducted in Kerala, Meghalaya, and Delhi. These states were chosen based on their: history of TCAM practice; the entrenchment and cultural consonance of certain systems of TCAM in their communities; differing administrative set-ups for the governance of AYUSH practice; and proximity to the centre of national policymaking in New Delhi. Kerala administers Ayurveda and Homoeopathy through distinct directorates, and does not co-locate AYUSH and allopathic practitioners. In contrast, in Delhi and Meghalaya, co-location of AYUSH and allopathic practitioners is common, although separate facilities for the different systems of medicine also exist. Certain AYUSH systems have an enduring presence in Kerala and Delhi, whereas several local healing traditions, such as Khasi and Garo medicine, rather than AYUSH systems, are inherent in Meghalaya (Albert and Porter, 2015).

### 2.2 Research approach

We applied an action-centred approach of policy implementation analysis (Barrett and Fudge, 1981, Hjern and Hull, 1982) in which policy implementation is regarded as a series of interactions and negotiations among actors, taking place in specific social and organizational contexts, seeking to distinguish policy as interpreted by relevant social actors, from the formal articulation of policies by state institutions (Hjern and Hull, 1982).

We employed two principal techniques of data-collection: in-depth interviews; and review of policy documents. In addition, researchers’ observations of the infrastructural arrangements and interpersonal interactions in the healthcare facilities were documented, and explored further in the interviews.

Reviewed policy documents included: stated national, state, and district policies for the mainstreaming of AYUSH; inter-office and intra-office memoranda on transfers, posting, in-service training, facilities, and grievances related to AYUSH personnel and supplies; and publicly available material on the internet. We mapped policy content using a framework developed for the assessment of governance architecture, functions, and policy and implementation gaps in an examination of regulation of healthcare in India (Sheikh et al., 2015).

Interviewees were drawn from a range of health policy and system actors involved in the mainstreaming of AYUSH, selected purposively based on principles of maximum variability (Silverman, 2001), in terms of age, occupation, area of expertise, years of work experience, type of employment, and geographical setting within study sites. Respondents were categorized as: key informants, including academicians, bureaucrats, and representatives of civil society organizations, with a deep understanding of the history and implementation of the inclusion of TCAM in the public health system of India; health system administrators, including state, district, and sub-district officials and supervisors at health facilities; TCAM (AYUSH and non-AYUSH) practitioners; allopathic doctors; and community representatives. In all, 196 interviews were conducted between February and October 2012. Table 1 enumerates the categories of participants across the study sites.

Interviews were audio-recorded with the respondents’ permission, and only notes taken when permission for audio-recording was not granted. The majority of the interviews were conducted in English, and some in a mixture of English and local languages.

### 2.3 Data analysis

Systematic content analysis of the policy documents was performed. Interviews were transcribed by research assistants, and checked for accuracy by the investigators. They were then analysed using the ATLAS.ti Version 7 software (Scientific Software Development GmbH). Data were processed using the thematic ‘framework’ technique that combines both inductive and deductive approaches (Ritchie and Spencer, 1994). *A priori* codes were generated, based on the interview guide, before data collection began. Cross-coding by at least two investigators was performed on a random selection of interviews, to ensure standardization in the application of codes. To the *a priori* codes were added emergent codes, dealing with meanings, values and rationale, developed jointly by the research team from studying the data. Analytical codes, corresponding to operational and ethical enablers and challenges in integration, were then jointly developed by the researchers from studying the patterning of emergent themes. Following coding, data were extracted, charted, and interpreted.

## 3 Results

This section describes the perceptions and expectations of the various health system actors of the role of an AYUSH provider, the dissimilar interpretations of work sharing of AYUSH practitioners and other health system actors, and the shortfalls in awareness of, and support for, AYUSH practice. Findings from this study relating to the facilitators and barriers of integration of TCAM providers in the public health system of India are reported elsewhere (Nambiar et al., 2014).

### 3.1 Discrepant role expectations

Role expectations varied by stakeholder category, as well as by region. Discrepancies among the perceptions of health system actors were greatest in regions where AYUSH providers were co-located with, and supervised by, allopathic providers, i.e., in Delhi and Meghalaya. In Kerala, where allopathic, Ayurvedic and Homoeopathic establishments were housed in separate physical facilities, and administered by distinct directorates, role expectations of AYUSH practitioners were not as much a matter of conflict. Table 2 summarises the various perceptions of the AYUSH provider's role held by the different stakeholders in the process of mainstreaming AYUSH in India.

#### 3.1.1 Policy articulations

For AYUSH practitioners appointed to government positions on either contractual or permanent bases, the national government mandated outpatient consulting in the respective AYUSH modality. Additionally, AYUSH providers were expected to participate in the national health programmes and administer the allopathic modules contained in the programmes, following training in these specific modules (Department of AYUSH, 2011). State governments, however, were authorised to elaborate on, and modify, the policy at their level. States’ interpretation, additions, and implementation led to dissimilar policies for AYUSH practice in different regions of the country (Dehury and Pattnaik, 2014). Thus, a few states and some districts within the same state, in contrast to other states or other districts from the same state, required one or more of the following activities from AYUSH providers, in addition to outpatient AYUSH consulting and participation in national health programmes: inpatient care in the respective AYUSH modality; participation in health camps, particularly in rural and remote areas; conducting childbirth; performing night duties; and performing emergency services at the health facility.

#### 3.1.2 AYUSH doctors’ expectations

AYUSH practitioners entered the public health workforce expecting to practise only their own system of medicine, mostly as outpatient consulting. Many expressed a desire to admit inpatients for AYUSH treatment, and some co-located facilities reported planning such an inclusion in their wards. Besides this, AYUSH providers expected to conduct the AYUSH component of the health camps organized by their facility, and, following training, to participate in national health programmes. Most expressed disinclination to take up components of allopathic practice, conscious of their lack of expertise in allopathic medicine, and their ineligibility to practice a system of medicine that they were not qualified in (an activity known as cross-practice). They reflected on how cross-practice would compromise the quality of care they could offer patients.

*“If they give me training in my field, I am open. I am comfortable in my field, right. I am not comfortable giving allopathic medicine. They want us to perform duties, like night duties, emergency, which I cannot handle … I won't do any justice to the patient, right. So I tried telling her* [allopathic supervisor] *this, but still it's very hard to sit down and have a talk.”* {AYUSH practitioner (contract)}

The following metaphor used to describe the role of AYUSH practitioners emerged in an interview, and encapsulates this perception:

“*… paani bharne wale se batti jalwana.”* [getting a water-carrier to light the lamps] {AYUSH practitioner (permanent)}

The AYUSH practitioner is analogized to a water-carrier, and the task of lamp-lighting is likened to the delivery of allopathic healthcare. This reflects expectations of service that are dissonant with the roles that AYUSH practitioners see themselves as having.

Some AYUSH providers were willing to learn and practise allopathic prescription in outpatient consulting, and perform night duties, and obstetric and emergency services. This was particularly true of those who did not have adequate provision for AYUSH practice, and had to share the premises of their allopathic colleagues. Moreover, some AYUSH providers expressed deep commitment to promoting community health, beyond prescribing medications, either allopathic or AYUSH, and engaged proactively in public health endeavours, such as family planning counselling.

*“For one year after I was posted here, I did not have any medicines. And I was sitting in the same room as the allopathic doctors. So, I used to help them … take cases and prescribe* [allopathic] *medicines.”* {AYUSH practitioner (contract)}

*“I believe that for us, OPD* [outpatient consulting] *is not as important. In the health sector, family planning is important … We have stayed up till 1 a.m., in villages, counselling people on no-scalpel vasectomy.”* {AYUSH practitioner (contract)}

AYUSH providers reflected on the different loyalties that they felt: to the welfare of patients; to the system of medicine that they were trained in; to the personnel team at the facility that they were appointed to; and to the country's public health. Many communicated frequent internal tussles among these loyalties, when inadequate provision for AYUSH practice, problematic communication, and interpersonal tension pulled them in different directions, unable to perform their roles as intended, and unwilling to compromise the care of the patient or the reputation of the professional team.

#### 3.1.3 Non-AYUSH TCAM providers’ expectations

Non-AYUSH TCAM practitioners, mostly local traditional healers, were largely outside the ambit of the government, national and regional, and not formally included as TCAM practitioners in the public health system, on either contractual or permanent bases.

*“There are local healing traditions that are part of the culture. To introduce, and more than that, to impose an alien system* [AYUSH systems in addition to allopathy] *is not right, when the traditional system is not being given any support.”* {Key informant}

In some regions, local healers were organized into associations that advocated their system of medicine, and pooled resources to provide care to the population in need. These practitioners distinguished AYUSH systems from the allopathic, as well as from their own healing practices. However, they did not necessarily distinguish among the different systems of AYUSH. They did not evince any expectations of the AYUSH provider's role in the public health system beyond AYUSH consulting.

#### 3.1.4 Allopathic doctors’ expectations

The activities expected by allopathic medical officers in charge of public health facilities, as the responsibilities of their AYUSH colleagues, varied across facilities, based largely on the volume of the inflow of patients and the capacity of the allopathic workforce at the facility to attend comfortably to it. In facilities with a low doctor-patient ratio, activities included, besides AYUSH consulting and participation in national health programmes and health camps: allopathic outpatient consulting; conducting childbirth; and performing night duties and emergency allopathic services, under remote supervision, i.e., in accordance with telephonically delivered advice from the allopathic supervisor. These expectations revealed the allopathic supervisors’ need for assistance rather than complementary practice. This need and its fulfillment were also expressed as approval of AYUSH practitioners known to take up and discharge health facility tasks other than AYUSH services voluntarily, as also in resentment of AYUSH practitioners reluctant to cross-practice.

*“See, some people love to work. Like the* [AYUSH] *doctor in* <name of a facility>*. Everyone is so happy with him. We want someone like him here.”* {Allopathic supervisor}

*“When they have a D-R in front of their names* [when they are called doctors]*, why can't they prescibe simple medicines like paracetamol and antibiotics? Even the staff nurse can do it, why can't they? We* [allopathic doctors] *are always available on phone to advise them.”* {Allopathic supervisor}

#### 3.1.5 Health system administrators’ expectations

In line with the state's and district's policy, health system administrators expected AYUSH personnel to engage in outpatient consulting in their respective AYUSH modalities, and additional work as specified, for which the district or state generally provided training. The reputation of individual AYUSH providers seemed to rely heavily on their enthusiasm for work other than AYUSH services at the health facility. AYUSH personnel who took up night duties, family planning counselling, obstetric services, and emergency services under allopathic supervision, and organized health camps, were appreciated widely.

*“He is the most faithful doctor. When the allopath is not there, he manages the whole PHC* [Primary Health Centre]*.”* {Health system administrator}

*“AYUSH doctors help with sterilization. Together* [with allopathic doctors]*, they motivate people.”* {Health system administrator}

On the other hand, some administrators decried the expectation of non-AYUSH services from AYUSH practitioners.

*“AYUSH doctors are not allowed to conduct deliveries.”* {Health system administrator}

*“AYUSH doctors can treat malaria patients only with pills, not injections. Complicated cases should be referred.”* {Health system administrator}

#### 3.1.6 Community members’ expectations

To a large extent, community members did not distinguish between allopathic and AYUSH doctors. This non-distinction was predominant in facilities where AYUSH supplies were low or absent, and the AYUSH provider helping with or engaging fully in allopathic practice. In some co-located facilities, the AYUSH providers had developed a reputation for effective treatment, enthusiasm, and cooperation with the facility staff, and had a high flow of patients in the AYUSH outpatient department. In other facilities, some community members were reported to request non-AYUSH procedures, such as injections of allopathic medications, from AYUSH providers.

### 3.2 Work sharing: complementing versus supplementing

All the interviewees evinced the understanding that sharing the work involved in ensuring population health was the rationale behind the purposive inclusion of AYUSH providers in the public health system of India. However, work sharing was interpreted variously by different stakeholders, the greatest contrast demonstrated between the views of the AYUSH practitioners on contracts, and their allopathic supervisors, administrative superiors, who were permanent employees. AYUSH practitioners expected to complement the work of their allopathic counterparts through AYUSH practice. However, some allopathic supervisors expected allopathic services (under supervision) from the AYUSH doctors to supplement their allopathic practice, and the latter felt the pressure to oblige, or the stress of resisting such expectations. The concept of integration emerged as one not understood uniformly by all the actors in the system: some considered the inclusion of personnel with AYUSH credentials sufficient (integration at the level of the practitioner, as it were), whereas others deemed that AYUSH had been included only when AYUSH was being practised by a qualified provider in the health facility (i.e., integration at the level of practice).

*“See, in our state, I think doctor population will be quite low. So inclusion of this thing* [AYUSH] *has reduced the load, the patient load, in the allopathic doctors.”* {Allopathic practitioner}

*“Eight AYUSH doctors are to be appointed for School Health. The government knows that they cannot get MBBS* [allopathic] *doctors easily.”* {Health system administrator}

*“I don't think he* [AYUSH doctor] *benefits from us, because we don't understand his drugs. We benefit because he helps us.”* {Allopathic practitioner}

### 3.3 Shortfalls in awareness of, and support for, AYUSH

Numerous circumstances of poor communication, preconceived notions, shortfalls in provision, and power imbalances have been reported in the medically pluralistic public health system of India. These form the context, and possibly the antecedents and influencers, of the discrepancies observed in the expectations of the roles of AYUSH providers.

#### 3.3.1 Low visibility for AYUSH providers

The roles of the AYUSH providers were ambiguously articulated, and very often not facilitated by the supplies, infrastructure, and personnel support required for optimal AYUSH practice. Some AYUSH providers reported that there was little or no communication of their appointment to the facility staff, including their assigned supervisors. There were practically no official channels for regular communication among practitioners and administrators of different systems of medicine. A few primary and community health centres reported a practice of plenary staff meetings, which gave the AYUSH practitioners and their facility colleagues the opportunity to interact with one another, and organize the allocation or sharing of infrastructure, personnel or other resources. There were some associations open only to AYUSH practitioners, with voluntary and discretionary membership, which facilitated deliberations, particularly on issues of recompense, supplies, and administrative hardships faced by the members, and helped convey these issues to the governing departments for resolution.

#### 3.3.2 Referral of patients based only on personal initiative

In co-located health facilities, there were no official procedures set up for cross-referral of patients and feedback on referred patients. AYUSH doctors reported referring cases that they deemed they could not handle, e.g., ‘emergencies’, to their allopathic counterparts. Referral of patients between practitioners of different systems of medicine was infrequent, and based entirely on personal initiative, often the patients', and the collegiality of the relationships among the staff within individual facilities. AYUSH practitioners who did not enjoy collegial relationships with their allopathic colleagues did not receive any referrals from them.

*“Informally patients are referred from allopathic treatment to Ayurvedic treatment, like for joint pains. We don't get much chance to interact with allopathy doctors. Patients themselves come here for treatment after allopathic treatment.”* {AYUSH practitioner (contract)}

In co-located facilities relatively free from logistical pressures, and with better formal and informal communication among the practitioners of different systems of medicine, patients were frequently cross-referred; practitioners discussed patients’ progress, and also sought treatment from one another, for themselves and their families.

#### 3.3.3 Low awareness of TCAM

Across all categories of interviewees, the majority expressed or demonstrated a lack of awareness of different systems of TCAM. This was true even of AYUSH practitioners, who generally exhibited unfamiliarity with systems of AYUSH other than their own. None of the interviewees displayed a nuanced understanding of the different systems of AYUSH. Several interviewees conflated the descriptions of different TCAM systems, including AYUSH, local health traditions, and home remedies. The acronym AYUSH, which stands for six distinct systems of medicine, was frequently confused with Ayurveda, one of the component systems, even by senior health system administrators.

*“One of our staff nurses took some AYUSH treatment … AYUSH or Homoeo, I am not sure.”* {Health system administrator}

This confusion was also evident, and transmitted to the public, in the boards put up at certain busy facilities, directing people to AYUSH consulting rooms, e.g., *“AYUSH and Homoeo department, third floor”.*

Furthermore, most health system administrators and allopathic supervisors had no knowledge of the medical curriculum and training of the AYUSH providers appointed in their facilities, and therefore, often underestimated or overestimated their scope. The unfamiliarity with different systems of AYUSH often went hand-in-hand with a low value assigned to AYUSH practices and practitioners.

*“Everybody thinks that AYUSH doctors cannot do this, cannot do that. But that is not the case. This doesn't feel good. We are seen with different eyes …. When we studied, there was a difference only in one subject. They studied pharmacology, and we studied materia medica. Every other subject was the same.”* {AYUSH practitioner (contract)}

*“We preach scientific medicine. They don't have proof. I don't know the basis of their medicines. We neither discourage nor interfere. Their medicines are effective – I am saying this from personal experience. I don't know much about its science. We never had much to do with them.”* {Allopathic practitioner}

*“Allopathic specialists can be given some orientation whereby they are made aware that these are the medicines in ISM* [Indian Systems of Medicine] *which you can prescribe. Not vice versa. An allopath is an ocean in herself. You cannot ask a pond to use the water of the ocean. You can ask the ocean.”* {Health system administrator}

#### 3.3.4 Disparities in provision and policy

In several facilities, the geographical and infrastructural arrangements made for AYUSH practice granted less visibility to the consulting rooms, and less convenience in physical access for patients. For instance, AYUSH consulting rooms were moved from the ground floor of a public hospital to the third floor, accessible only through a stairway from a screened corridor, with the sole direction to this section being a hand-written note pasted on a wall next to the stairway. In a rural location, the AYUSH section was placed in a new building, with no directions posted at the main building. The mismatch between infrastructural provision and role requirements of AYUSH practitioners was cited by several study participants.

AYUSH providers were seldom at the helm of organizations, or of individual public health facilities, except in regions where they were placed in exclusively AYUSH facilities or departments. AYUSH providers appointed to positions in hospitals and those appointed to various other public health clinics as contractual staff had supervisors who were allopathic doctors. Supervision took the form of documentation of attendance and channelizing of indents, requests, and official communications through the supervisor to administrative superiors. Allopathic supervisors and AYUSH providers working within hierarchical reporting structures, in the milieu of partial information and, frequently, disciplinary biases, often found themselves in strained, unfulfilling, and stressful working conditions.

There were glaring budgetary and policy disparities between support for AYUSH systems on the one hand and the allopathic system on the other. Policymaking for AYUSH in India was not perceived as adequately participatory, and sufficiently responsive to the nature of the AYUSH systems, the needs of the personnel, and the preferences of the population. In fact, AYUSH, although officially a department in the Ministry of Health and Family Welfare, was often treated as an outside entity at key moments of planning and decision-making, even decision-making for AYUSH personnel. AYUSH providers described numerous lacunae in the provision of supplies and human resources support, such as the egregious delays and shortfalls in the supply of medicines, and the inconvenient elimination of the role of a technical assistant to help with clinical consultations and dispense prescriptions. Health system administrators and key informants also remarked on the low support for research in AYUSH, a situation presenting bleak prospects for the development of a strong evidence base for the efficacy of the various AYUSH systems.

*“These policies are made by health people* [the Department of Health and Family Welfare]*, and AYUSH people are asked later.”* {AYUSH practitioner (permanent)}

*“To function well, we need to have someone to dispense medicines, and at least one male and one female assistant to do panchakarma and massage, etc. We don't have anyone now. The post of assistant has been removed.”* {AYUSH practitioner (permanent)}

*“Our colleges are not as strong. There's only enough money for salaries in the Research Councils, we need to fix this.”* {Health system administrator – AYUSH }

## 4 Discussion

This study, undertaken to explore operational and ethical challenges in the integration of AYUSH providers in the public health system of India, had the methodological strengths of a review of policy; in-depth interviews with a wide range of health system actors in three geographically distant, and administratively and culturally different states of India, with varying levels of indigeneity and entrenchment of different TCAM systems in the community; and observations and analysis by a team of researchers. The elicitation of participants' experiences and opinions, as well as the interpretation of the findings could have been limited by the researchers’ expertise and viewpoints.

A major finding of this study was discrepant perceptions of the role of the AYUSH provider in government health facilities. National policy articulations closely matched AYUSH providers’ expectations of their role descriptions. In contrast, the perceptions held by health system administrators, and allopathic counterparts and supervisors differed, often greatly, from the perceptions of the AYUSH providers. Discrepant role expectations emerged as the nub of the interpersonal tension among health system actors in the integration of AYUSH into the public health system. These discrepancies had the most adverse impact in Meghalaya and Delhi, where co-location of practitioners of different systems of medicine was in operation, and, within these states, in the facilities shared by practitioners of different systems of medicine, placed in hierarchical administrative structures. In Kerala, where practitioners of different systems of medicine were not co-located, tensions related to discrepant role expectations did not play out as much in interactions and day-to-day functioning, although numerous other divergent perceptions of the different systems of medicine were expressed. Another context in which the discrepancies in perception were expressed was the variable entrenchment and indigeneity of the various AYUSH systems in the states studied. Thus, AYUSH was often confused with Ayurveda in Meghalaya, but not in Kerala, where people were much more familiar with Ayurveda. Local health traditions played a more prominent role in the discourse in Meghalaya than in the other states.

Gaps in role descriptions for personnel, as well as in the articulation of referral and collaboration protocols at healthcare facilities (Chandra, 2011, SEDEM, 2010, Priya and Shweta, 2010, Gopichandran and Kumar, 2012, Priya, 2013) resulted in most of the AYUSH providers’ functioning depending upon logistical provision, directions from supervisors, and personal initiative. Studies in other countries and settings, among different cadres of health workers, have also described adverse impacts of role conflict, role ambiguity, and difficulty working with other professional groups; and of organizational conditions, health facility environment, and inadequate logistical provision on the satisfaction, practices, and performance of health workers (Rowe et al., 2005, Acker, 2004, Drolen and Harrison, 1990).

The most contentious of the expectations held about AYUSH providers’ functions revolved around cross-practice, specifically the practice of allopathic medicine by AYUSH providers. Participants in our study displayed a range of stances towards cross-practice: Certain allopathic supervisors of AYUSH practitioners in co-located facilities expected and encouraged allopathic practice by the AYUSH practitioners. AYUSH practitioners were mostly reluctant, or, in some cases, frankly opposed to prescribing allopathic medications, while a few were quite willing to do so. Administrators and academic experts, as well as several practitioners in this study, did not advocate cross-practice.

Some proponents of cross-practice recommend the prescription of allopathic medications by AYUSH practitioners for medical emergencies, and for situations in which AYUSH medications are unavailable (Ravishanker, 2014). Singh and Raje (1996) noted that people's observation of TCAM practitioners prescribing allopathic medicine marred the reputation of the TCAM systems that the practitioners were qualified in. Sharma (2001) pointed out that exposure of doctors to systems of medicine other than the one they were qualified in could engender the risk of cross-practice, and quackery. Cross-practice has been considered a potential threat to population health as it is likely to compromise safety and quality of care, and has, therefore, usually been disallowed in national and state policy (Dar et al., 2015), with some exceptions in certain states of India (Press Information Bureau, 2013, Priya, 2013). These exceptions, in turn, have been fraught with controversy among practitioners, administrators, and beneficiaries. For instance, allopathic professional associations denounced government policies that permit AYUSH practitioners to prescribe and administer allopathic medication (IMA, 2014, Pillai and Aggarwal, 2014), whereas certain AYUSH associations and public health administrators viewed such policies as increasing the accessibility of health services among the public (The Indian Express, 2015), enlarging the scope of AYUSH providers' therapeutic repertoire, and curbing quackery (Yasmeen, 2013). In light of the minimal, if any, education and training that practitioners of several AYUSH systems have in allopathic medicine, the expectation that AYUSH practitioners employed in public health facilities practise allopathic medicine, either to substitute for their allopathic colleagues or to supplement their allopathic colleagues' work has been seen to go against the greater interests of public health (Gopichandran and Kumar, 2012), as well as the prospect of the development of the fields of AYUSH (Dar et al., 2015). The converse situation, of allopathic practitioners prescribing AYUSH medications, although similarly risky and unethical, has rarely come up in public health facilities in India, and no expectation of AYUSH prescriptions from allopathic practitioners has been reported.

Several researchers have commented on the hegemony of allopathic medicine across the world (Jefferey, 1982, Naraindas, 2006, Sujatha, 2011, Priya, 2012), and the support for the perpetuation of this hegemony that comes from policy, administration, and budget disparities. For instance, Chi (1994) reported low participation of Chinese medicine practitioners in public health policymaking in Taiwan. Ngetich (2008) commented on the stark dissonance between the budgetary allocations for the traditional and modern systems of medicine, and the prevalence of their use by the population, specifically the very low allocation of health system resources to traditional medicine, in the face of the high prevalence of use of traditional medicine. Further, political and administrative decisions such as the positioning of traditional medicine in the ministry of culture rather than the ministry of health have been seen to deprive the traditional medicine sector of full recognition as a scientific set of practices, and of the benefits of financial protection, such as health insurance; and to militate against efficient and transparent governance of the medically pluralistic health system (Ngetich, 2008). Shukla and Gardner (2006) recognized the inadequacy of support for TCAM in India, and further, the danger of steadily declining popular interest in TCAM and the threat of extinction of unsupported TCAM systems. The proportion of the total budget for health that has been allocated to AYUSH has been as low as 1.3 to 2.7 percent over the past decade (Priya, 2012). The experiences of our study participants echo these observations of problematic administrative hierarchies, budgetary lacunae, and exclusion from decision-making roles for TCAM practitioners. The highly discrepant allocations for the allopathic and AYUSH sectors, from the budget for healthcare, have clear adverse consequences for the AYUSH fields, as demonstrated in the lower research output, lower institutional provision for education and clinical care, and lower remuneration for personnel (Dar et al., 2015, Chandra, 2011, Priya, 2012, Lakshmi, 2012). This study also confirms other researchers’ findings that notwithstanding the sporadic policy articulations to extend support to local health traditions, local health traditions continue to be marginalized by the public health system (Albert and Porter, 2015, Priya, 2013).

Gaps in infrastructural provision and supplies, including inadequacy of space, storage facilities, signage, medications, and office supplies, which reports over the years show have dogged AYUSH practices in mainstreaming endeavours (Dar et al., 2015, SEDEM, 2010, Chandra, 2011, Priya and Shweta, 2010, Lakshmi, 2012, Dehury and Pattnaik, 2014) were reported by virtually every AYUSH practice established on a contractual basis in public health facilities, in this study. AYUSH practitioners also stressed the unmet need for human resources support, specifically a trained assistant and a compounder, for their practice.

Studies conducted in integrated practices of biomedicine and TCAM in other countries (Hollenberg, 2006, Jingfeng, 1988) have revealed attitudinal barriers to communication and congenial coexistence among allopathic and TCAM practitioners. These studies shed light on certain official procedures, such as restricted access to patient-charts for TCAM practitioners, and restrictions on patient-referral (Hollenberg, 2006), that placed constraints on the TCAM practitioners’ practice, as well as the absence or dearth of official procedures that facilitate regular communication for collaboration among the practitioners. In Kenya, the lack of acknowledgement and provision for the fact that patients choose to take recourse to multiple systems of medicine was demonstrated in the absence of formal or informal platforms for practitioners of different systems of medicine to exchange views and collaborate, as well as the absence of protocols for cross-referral (Ngetich, 2008).

Other examinations of the functioning of co-located AYUSH and allopathic facilities have found little or no interaction, professional or social, among providers practising different systems of medicine at the same facility, no published protocol for cross-referral (Shrivastava et al., 2015, Chandra, 2011, Priya, 2013), little or no cross-referral (SEDEM, 2010), and no documentation of referral when it does occur (Priya and Shweta, 2010, Gopichandran and Kumar, 2012, Priya, 2013).

Communication between AYUSH providers and allopathic peers and supervisors in our study was often reported to be minimal, fraught with tension, or even avoided if possible (SEDEM, 2010), although some instances of collegial relationships among providers practising different systems of medicine at shared facilities were also observed. The power differential, engendered by the hierarchical reporting structures in the public health system, likely underpinned this tension (Priya, 2013). Our study did not reveal any official procedural barriers to the participation of practitioners of different systems of medicine in the care of patients. However, the absence of formal protocols for cross-referral of patients, and the scarcity of platforms such as organization-wide meetings and institutional events for formal and informal interaction led to referrals and interaction occurring only on the personal initiative of the providers, predicated on the organizational culture at each individual health facility.

### 4.1 Implications for a pluralistic public health system

The role ambivalence of TCAM (AYUSH and non-AYUSH) practitioners, and the contestations among health system actors over the functions and contributions expected of TCAM practitioners, have implications for TCAM practice, TCAM provider morale, and overall health system efficiency and performance. The low policy and budgetary support for TCAM, as well as the logistical pressures upon TCAM practice, in addition, pose the threat of the loss of the integrity of individual TCAM systems in a notionally integrated health system, and the possible loss of bodies of TCAM knowledge and praxis. Interpreting ‘integration’ as a reclassification of TCAM techniques and products into biomedical categories, without the concepts underlying their use, has also raised concerns among researchers and advocates of medical pluralism (Sujatha, 2011, Naraindas, 2006).

### 4.2 Strategic directions and recommendations for future work

Potential directions suggested by the findings of this study include unambiguous articulation of the roles and responsibilities of the TCAM providers appointed to public health facilities in medically pluralistic systems. In addition, clear delineation of the procedures to be followed in situations of delayed or inadequate infrastructural provision, supplies, and personnel support would be helpful. The provision of basic information to the clinical and support staff, on the system of TCAM to be practised at each facility, would aid in establishing peaceful coexistence of different systems of medicine at the facility, and help more patients access TCAM practitioners with ease. A regular and transparent routine of plenary staff meetings, and the promotion of professional associations to discuss strategies and solutions, would help keep communication channels open.

Broader health system related recommendations to improve role clarity are of improved communication, referral protocols, and awareness-raising. Financial and political support, such as the revision of the budgetary allocation to the TCAM sector at national, state and district levels, to provide adequate support for education, research, community outreach, infrastructure and supplies, and staffing and training, are crucial to steer the public health system towards vibrant and effective pluralistic healthcare.

Further work is called for on ascertaining the awareness and attitudes among health system actors regarding the different systems of medicine in practice in their communities. Future studies could also examine policymaking, and provision, for the practice of various TCAM systems in a medically pluralistic health system.

## Figures and Tables

**Table 1:** Categories of interviewees.

	Kerala	Meghalaya	Delhi	Total
Key informants	2	3	7	**12**
Health system administrators	16	13	14	**43**
AYUSH doctors	27	14	19	**60**
Non-AYUSH TCAM providers	0	6	0	**6**
Allopathic doctors	13	13	11	**37**
Community representatives	16	12	10	**38**
Total	**74**	**61**	**61**	**196**

**Table 2:** Discrepant perceptions of the role of an AYUSH provider.

Role of AYUSH provider	Stakeholder						
	National govt.	State govt.s	AYUSH doctors	Non-AYUSH TCAM providers	Allopathic doctors	Health system administrators	Community members
Outpatient consultation – allopathic					*√*^a^		*√*^a^
Outpatient consultation – AYUSH	*√*	*√*	*√*	*√*	*√*	*√*	*√*
Inpatient consultation – AYUSH	*√*	*√*	*√*	*√*	*√*	*√*	*√*
National health programmes	*√*	*√*	*√*		*√*	*√*	*√*
Health camps (family planning)					*√*		
Health camps (AYUSH)	*√*	*√*	*√*	*√*	*√*	*√*	*√*
Night duties					*√*^a^	*√*^a^	
Emergency services					*√*^a^	*√*^a^	
Conducting childbirth		*√*^a^			*√*^a^		

a In some, not all, healthcare facilities
